# Antibiotics: Combatting Tolerance To Stop Resistance

**DOI:** 10.1128/mBio.02095-19

**Published:** 2019-09-10

**Authors:** Etthel M. Windels, Joran E. Michiels, Bram Van den Bergh, Maarten Fauvart, Jan Michiels

**Affiliations:** a VIB Center for Microbiology, Flanders Institute for Biotechnology, Leuven, Belgium; b Centre of Microbial and Plant Genetics, KU Leuven, Leuven, Belgium; c imec, Leuven, Belgium; Northeastern University; Harvard School of Public Health

**Keywords:** antibiotic resistance, antibiotics, evolution, persistence

## Abstract

Antibiotic resistance poses an alarming and ever-increasing threat to modern health care. Although the current antibiotic crisis is widely acknowledged, actions taken so far have proved insufficient to slow down the rampant spread of resistant pathogens. Problematically, routine screening methods and strategies to restrict therapy failure almost exclusively focus on genetic resistance, while evidence for dangers posed by other bacterial survival strategies is mounting. Antibiotic tolerance, occurring either population-wide or in a subpopulation of cells, allows bacteria to transiently overcome antibiotic treatment and is overlooked in clinical practice. In addition to prolonging treatment and causing relapsing infections, recent studies have revealed that tolerance also accelerates the emergence of resistance. These critical findings emphasize the need for strategies to combat tolerance, not only to improve treatment of recurrent infections but also to effectively address the problem of antibiotic resistance at the root.

## Abstract (precis)

Antibiotic resistance poses an alarming and ever-increasing threat to modern health care. Although the current antibiotic crisis is widely acknowledged, actions taken so far have proved insufficient to slow down the rampant spread of resistant pathogens. Problematically, routine screening methods and strategies to restrict therapy failure almost exclusively focus on genetic resistance, while evidence for dangers posed by other bacterial survival strategies is mounting.

## ANTIBIOTICS AND THE RESISTANCE CRISIS

Antibiotics have transformed medicine and saved millions of lives since the introduction of penicillin in the 1940s (1). However, either by *de novo* mutation or horizontal gene transfer, bacteria can acquire mechanisms to degrade or inactivate the antibiotic, pump the antibiotic out of the cell, or modify the drug target. These resistance mechanisms allow bacteria to grow at elevated concentrations of antibiotics. Reports on resistance to antibiotics appeared even before their first therapeutic use (2). Initially, the emergence of resistant pathogens was not a matter of significant public concern, as novel effective antibiotics were being discovered regularly. However, after the golden years of antibiotic discovery from the 1940s through the 1960s, the development of new and effective antibiotics steadily declined (3). The decreasing supply of novel compounds, together with the massive increase in antibiotic consumption, including nontherapeutic use for growth promotion in agriculture and aquaculture, has led to the antibiotic resistance crisis that we are facing today (4). Each year, infections with antibiotic-resistant bacteria are estimated to result in more than 23,000 and 33,000 deaths in the United States and Europe, respectively (5, 6). Infections with antibiotic-resistant pathogens cause an extra 10,000 to 40,000 U.S. dollars in hospital costs per patient (7). If adequate measures are not taken, the toll of antimicrobial resistance in Europe, North America, and Australia is estimated to rise to 2.4 million casualties per year at an annual cost of up to 3.5 billion U.S. dollars (8).

## ANTIBIOTIC TOLERANCE AND PERSISTENCE

Overshadowed by the more prominent narrative of antibiotic resistance, scientists have also warned of the dangers of another strategy by which bacteria can overcome antibiotic treatment. Antibiotic tolerance allows bacteria to temporarily withstand or slow down the lethal consequences of high doses of bactericidal antibiotics but without being able to grow in their presence (9). Increased tolerance can result from mutations, but it can also result from environmental conditions, for example conditions that slow down growth (10). The ability of a whole bacterial population to survive longer treatments with bactericidal antibiotics is denoted as “tolerance” throughout the text. Tolerance can also occur in a subpopulation of phenotypic variants called “persister cells.” This specific type of tolerance is referred to as “persistence” (11). As was the case with resistance, tolerance and persistence were first observed shortly after the introduction of penicillin (12–15).

Increasing evidence suggests that tolerance and persistence play a considerable and currently underappreciated role in the recalcitrance and relapse of bacterial infections (11, 16–19). This evidence includes the following. (i) High levels of persisters are present in biofilms and inside host cells (20–23). (ii) A well-known persistence gene is frequently mutated in clinical isolates of uropathogenic Escherichia coli (24). (iii) Increased tolerance or persistence evolves in the face of periodical antibiotic treatment both *in vitro* and *in vivo* (25–29). (iv) Recurrent episodes of infection can be due to relapse caused by the original, nonresistant founder strain rather than reinfection with a new strain (30–32). (v) Several studies demonstrated that nongrowing or slowly growing bacteria tolerate antibiotics and cause therapy failure in *in vivo* infection models (23, 33–37). The increasing awareness of the clinical consequences of tolerance and persistence has strongly encouraged research on these phenomena in the past decade. However, different experimental setups are often adopted in different studies, resulting in inconsistencies in the available data. A set of standardized and generally approved guidelines were recently proposed to measure antibiotic persistence and should facilitate the comparison of experimental results (38).

## TOLERANCE AND PERSISTENCE AS DRIVERS OF RESISTANCE EVOLUTION

Three decades ago, Moreillon and Tomasz found that cyclic exposure of Streptococcus pneumoniae to high concentrations of penicillin selects for tolerant mutants, while resistant mutants evolve during exposure to sustained, low levels of penicillin (39). On the basis of this finding, they hypothesized that tolerant cells constitute a reservoir of viable cells from which resistant mutants can emerge (“Resistant mutants would then be recruited from this pool of lysis- and kill-defective bacteria […]”), implying that tolerance facilitates the evolution of resistance (“selection for resistance […] may be secondary to selection for increased survival.”). In further agreement with this hypothesis, a tolerant S. pneumoniae mutant consistently displays greater efficiency in transformation with DNA from streptomycin- or penicillin-resistant clinical isolates compared to a wild-type S. pneumoniae strain (40). Mathematical simulation of a bacterial infection similarly showed that tolerant cells may play an important role in the emergence of therapy resistance (41). However, strong experimental evidence in favor of this hypothesis was published only recently. Nguyen et al. used a murine infection model to demonstrate that resistance development is abolished in a strain with strongly decreased antibiotic tolerance (21). Sebastian et al. showed that persisters are a source of *de novo* resistant mutants during long-term rifampin exposure in Mycobacterium tuberculosis (42). The group of Nathalie Balaban found that the evolution of increased tolerance or persistence precedes the emergence and spread of resistance-conferring mutations in E. coli populations under intermittent ampicillin exposure (43). Recently, we discovered a strong, positive correlation between persister levels and the likelihood to evolve resistance in natural isolates and lab strains of E. coli (44). Importantly, these findings hold for different types of antibiotics, in different setups or conditions, suggesting a widespread link between persistence and the evolution of resistance.

Apart from constituting a viable reservoir from which resistant clones can emerge, tolerance and persistence may also facilitate the development of resistance in a more intricate fashion. Stress responses play a central role in the formation of persisters (11, 16, 21, 45), and are also known to cause a temporal increase in cellular mutation rates (46–49). Hence, high persister levels and high mutation rates may act synergistically in stressed bacteria and increase the likelihood for resistance-conferring mutations to occur in the persister reservoir (50). Evidence for stress responses being a major determinant of the persistence-resistance link was provided by Sebastian et al., who reported that resistance mutations occurring in *Mycobacterium* persisters are provoked by elevated levels of oxidative stress (42). Moreover, high-persistence and mutator phenotypes are both known to thrive in fluctuating environments (25, 51–53), suggesting that these traits could evolve under similar environmental conditions. A recent study from our lab confirmed that persistence is pleiotropically linked with mutation rates. Using Luria-Delbrück fluctuation assays, we found increased mutation rates in two high-persistence mutants and a lower mutation rate in a low-persistence strain. In populations plated on supra-MIC antibiotic concentrations, the number of resistant mutants that emerged per surviving persister shows the same trend (44). Spontaneous or antibiotic-induced DNA damage may still inflict mutations in nongrowing persisters, because these cells display DNA turnover even in the absence of chromosomal replication (54–56). Indeed, Barrett et al. recently observed a strong induction of the SOS response in persisters, indicative of DNA damage (57). These persisters exhibit accelerated resistance development, a process in which error-prone DNA polymerases are involved (57). Similarly, Yaakov et al. demonstrated that Saccharomyces cerevisiae persisters are characterized by an increased load of DNA damage, pointing at an increased mutation rate and evolvability of these cells (58). A correlation between persistence and mutation rates was also recently confirmed by El Meouche and Dunlop (59). Their data suggest that heterogeneity in the expression of the multidrug efflux pump AcrAB-TolC gives rise to a subpopulation that is not only transiently resistant to several drugs but also exhibits a lower growth rate and a reduced expression of the DNA mismatch repair enzyme MutS, resulting in an increased spontaneous mutation rate (59).

**FIG 1:**
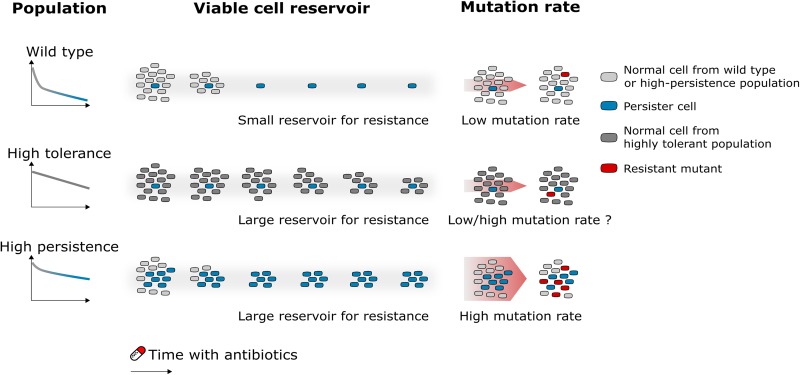
Framework for how antibiotic tolerance and persistence accelerate the evolution of genetic resistance. First, in populations displaying high levels of tolerance or persistence, an increased number of viable cells is available for mutation, increasing the likelihood for a resistant mutant to arise. Second, persistence is linked with higher mutation rates, again causing an increased likelihood for the occurrence of resistance-conferring mutations. Future research will reveal if a similar link exists between tolerance and mutation rates.

On the basis of this accumulating experimental evidence, we propose a framework in which two factors constitute the link between tolerance or persistence and resistance (Fig. 1). On the one hand, high levels of persistence or tolerance lead to a higher number of viable cells during antibiotic treatment, which results in an increased statistical probability for the occurrence of resistance-conferring mutations (Fig. 1). On the other hand, increased persistence, and possibly also tolerance, is pleiotropically linked with increased mutation rates both in growing cells (when antibiotic concentrations are low) and in persisters (when antibiotic concentrations are high) (Fig. 1). A mathematical model that simulates the course of antibiotic treatment in a patient with a bacterial infection confirmed that persistence contributes to the emergence of resistance in clinical settings by an interplay of increased survival and increased adaptability through elevated mutation rates (44).

## COMBATTING TOLERANCE TO STOP RESISTANCE

Continuous improvements in medical care increase the life span of immunocompromised patients, and the use of indwelling medical devices, prone to biofilm-related infections that shield bacteria from the activity of the immune system, is ever growing. Therefore, the clinical manifestation of antibiotic tolerance can be expected to increase considerably in the future. Given the recent studies that unravelled a link between tolerance or persistence and the evolution of genetic resistance, this will lead not only to an increased prevalence of chronic and recurrent infections but likely also to a further increase in the emergence and spread of antibiotic resistance. Therefore, to avoid the devastating consequences of the imminent postantibiotic era, we argue that the clinical focus should not lie exclusively with battling antibiotic resistance.

In clinical settings, screening for resistance by disk diffusion assays or MIC measurements has become routine practice to guide antibiotic therapy of bacterial infections (60). On the other hand, antibiotic tolerance and persistence are overlooked, mainly due to the lack of a similarly reliable, quantitative, and easily measurable parameter. As a consequence, data on the clinical manifestation of tolerance and persistence are rather scarce (9). On the basis of the decreased killing rate characterizing antibiotic-tolerant cells, the Balaban group introduced the “minimum duration for killing” (MDK) as a quantitative parameter to identify tolerance and persistence (28, 61). As time-kill assays are labor-intensive, the same group also described a modification of the standard disk diffusion assay that allows a semiquantitative evaluation of tolerance and persistence (62). However, further validation of these proposed metrics in species other than E. coli and in clinical isolates is necessary before they can be implemented by clinical microbiology labs. Moreover, it is important to note that tolerance and persistence strongly depend on the environmental conditions, necessitating their detection to occur in a context that resembles the *in vivo* infection environment (63). Current attempts to investigate this *in vivo* context include the use of engineered strains reporting on the environmental conditions and their impact on bacterial physiology (64, 65). This knowledge should be used to move away from conventional culture conditions toward model systems that mimic the infection environment (65).

Therapeutic choices could be further supported by sequence-based prediction of resistance and tolerance. The ever-decreasing costs of sequencing make the identification of tolerance- and persistence-associated genetic markers increasingly feasible (18). Nevertheless, knowledge gaps regarding the genetic basis of tolerance and persistence are currently hampering the use of DNA-based diagnostic tools. On the other hand, expression-based and physiology-based markers have been proposed as tools to diagnose antibiotic tolerance and persistence (18, 66). For example, Stokes et al. recently developed the “solid media portable cell killing” (SPOCK) assay, a method that allows high-throughput screening of antibiotic killing of tolerant bacteria (67). In this assay, antibiotic lethality against cells residing in colonies is monitored with a redox-sensitive dye (67). However, this method does not assess the capacity for regrowth of surviving cells, and therefore does not discriminate between antibiotic-tolerant cells and viable but nonculturable cells (VBNCs). As the existing methods to diagnose tolerance and persistence are clearly still in their infancy, we advocate that more research should be focused on the further development and valorization of these tools. A better understanding of the genetic basis and physiology of antibiotic-tolerant cells would improve the prospect of marker-based diagnostics. Furthermore, an expeditious introduction of these tools in screenings for novel antibiotics and clinical decision-making will become indispensable.

In addition to the development of diagnostic tools for tolerance and persistence, considerable efforts should be devoted to the development of strategies able to eliminate tolerant cells, as these have the potential to preclude the evolution of resistance. The effectiveness of this approach was illustrated in a proof-of-concept experiment where E. coli cultures were treated with mannitol. This compound is known to sensitize persister cells, and consequently slows down resistance development (44). Similarly, it has been proposed that inhibitors of the AcrAB-TolC efflux pump might not only sensitize cells to antibiotics but also restore their mutation rates, potentially leading to an improved treatment outcome (68).

Strikingly, drug-tolerant persisters are also present in cancer cell populations, where they are implicated in tumor recurrence (69, 70). An increasing body of evidence suggests that this subpopulation of phenotypic variants acts as a substantial reservoir for the emergence of therapy resistance (71, 72). This intriguing parallel between cancer and infections, two seemingly distinct types of disease, indicates that antipersister strategies may also help to improve the treatment outcome of cancer (73–75). Indeed, inhibition of the lipid hydroperoxidase GPX4, which is necessary for survival of persisters, results in persister cell death and consequently prevents the acquisition of drug resistance by cancerous cells (76). Understanding, detecting, and targeting tolerance and persistence will require joint efforts of microbiologists and clinicians and should eventually lead to reduced therapy failure in both infectious diseases and cancer.
